# MicroRNA-223 is a novel negative regulator of HSP90B1 in CLL

**DOI:** 10.1186/s12885-015-1212-2

**Published:** 2015-04-08

**Authors:** Ana E Rodríguez-Vicente, Dalia Quwaider, Rocío Benito, Irena Misiewicz-Krzeminska, María Hernández-Sánchez, Alfonso García de Coca, Rosa Fisac, José-María Alonso, Carolina Zato, Juan Francisco de Paz, Juan Luis García, Ma Eugenia Sarasquete, José Ángel Hernández, Juan M Corchado, Marcos González, Norma C Gutiérrez, Jesús-María Hernández-Rivas

**Affiliations:** 1 Servicio de Hematología, IBSAL, IBMCC, CIC, Universidad de Salamanca, CSIC, Hospital Universitario, Salamanca, Spain; 2 National Medicines Institute, Warsaw, Poland; 3 Servicio de Hematología, Hospital Clínico Universitario, Valladolid, Spain; 4 Servicio de Hematología, Hospital General de Segovia, Segovia, Spain; 5 Servicio de Hematología, Hospital Río Carrión, Palencia, Spain; 6 Departamento de Informática y Automática, Universidad de Salamanca, Salamanca, Spain; 7 Instituto de Estudios de Ciencias de la Salud de Castilla y León, (IECSCYL)–HUSAL, Castilla y León, Spain; 8 Servicio de Hematología, Hospital Universitario Infanta Leonor, Universidad Complutense de Madrid, Madrid, Spain

**Keywords:** Chronic lymphocytic leukemia, MicroRNAs, Next-generation sequencing

## Abstract

**Background:**

MicroRNAs are known to inhibit gene expression by binding to the 3′UTR of the target transcript. Downregulation of miR-223 has been recently reported to have prognostic significance in CLL. However, there is no evidence of the pathogenetic mechanism of this miRNA in CLL patients.

**Methods:**

By applying next-generation sequencing techniques we have detected a common polymorphism (rs2307842), in 24% of CLL patients, which disrupts the binding site for miR-223 in *HSP90B*1 3′UTR. We investigated whether miR-223 directly targets HSP90B1 through luciferase assays and ectopic expression of miR-223. Quantitative real-time polymerase chain reaction and western blot were used to determine HSP90B1 expression in CLL patients. The relationship between rs2307842 status, *HSP90B1* expression and clinico-biological data were assessed.

**Results:**

HSP90B1 is a direct target for miR-223 by interaction with the putative miR-223 binding site. The analysis in paired samples (CD19+ fraction cell and non-CD19+ fraction cell) showed that the presence of rs2307842 and IGHV unmutated genes determined *HSP90B1* overexpression in B lymphocytes from CLL patients. These results were confirmed at the protein level by western blot. Of note, *HSP90B1* overexpression was independently predictive of shorter time to the first therapy in CLL patients. By contrast, the presence of rs2307842 was not related to the outcome.

**Conclusions:**

*HSP90B1* is a direct target gene of miR-223. Our results provide a plausible explanation of why CLL patients harboring miR-223 downregulation are associated with a poor outcome, pointing out *HSP90B1* as a new pathogenic mechanism in CLL and a promising therapeutic target.

**Electronic supplementary material:**

The online version of this article (doi:10.1186/s12885-015-1212-2) contains supplementary material, which is available to authorized users.

## Background

MicroRNAs (miRNAs) are endogenously expressed small RNA molecules that mediate posttranscriptional gene silencing through complimentary binding of the 3′untranslated regions (3′UTR) of target genes [1]. Over half of the human transcriptome is predicted to be under miRNA regulation, embedding this post-transcriptional control pathway within nearly every biological process [2-4]. Thus, miRNAs are involved in almost all aspects of cancer biology, such as proliferation, apoptosis, invasion/metastasis, and angiogenesis [5].

Over the past few years several studies have shown that miRNAs play an important role in CLL [6-9]. Distinct microRNA signatures are associated with prognosis, disease progression [9-14] and response to treatment [15,16]. In CLL, the downregulation of miR-223 is associated with disease aggressiveness and poor prognostic factors [13,14], which may become this miRNA a new reliable prognostic predictor. However, unlike other miRNAs with prognostic value in CLL such as miR-181b and miR- 29c, there is no evidence of its pathogenetic role, and no target has so far been proposed or validated for miR-223 in CLL.

Over the last decade, several studies have implicated heat shock proteins (HSPs) as major contributors to cancer progression and the development of chemoresistance. HSPs are upregulated in many cancers, including CLL, and may contribute to prolonged tumor cell survival via several mechanisms that remain to be fully described [17-19]. Preclinical studies in CLL have shown that *HSP90* inhibition causes the degradation of ZAP-70 and other proteins associated with poor survival, and this may ultimately lead to apoptosis [20-24]. Targeting *HSP90* is an attractive strategy in CLL as this could represent a therapeutic option to drug resistance in CLL associated with lesions in the *ATM/TP53* pathway [25-27]. Thus, inhibitors of *HSP90* have been proposed as a novel therapeutic option for CLL [28-30]*.*

By applying next-generation sequencing (NGS) techniques we have detected a common polymorphism (rs2307842), in 24% of CLL patients, which disrupts the binding site for miR-223 in *HSP90B*1 3′UTR, leading to its overexpression in clonal B lymphocytes. This finding has helped us to identify miR-223 as a regulator of HSP90B1 levels in CLL patients, with therapeutic consequences.

## Methods

### Patients and controls

Four patients with CLL were selected for a Targeted Sequence Capture and DNA Sequencing assay. CLL diagnosis was performed according to World Health Organization (WHO) classification [31] and Working Group of National Cancer Institute (NCI) criteria [32]. CD19+ fraction cells were used for sequencing and were obtained before administration of any treatment. To determine the clinical impact of *HSP90B1* 3′UTR polymorphism, we expanded the study to 165 additional patients with CLL and 32 healthy controls. FISH studies and *IGHV* mutational status were assessed. Details on the main characteristics of the 169 CLL patients included in the study are reported in Table 1 and Additional file 1: Supplementary Methods. The study was approved by the local ethical committee “Comité Ético de Investigación Clínica, Hospital Universitario de Salamanca”. Written informed consent was obtained from each patient before they entered the study.

**Table 1:** **Clinical and biological features of the CLL patients included in the study**

Parameter	Category	
Age (years), median (range)		66 (34-90)
Gender	Male	66.0%
White blood cells/mL (range)		21 545 (7 080-188 020)
Lymphocytes/mL (range)		15 741 (1 580-180 000)
Hemoglobin, g/dL (range)		14.1 (4.4-16.8)
Platelet count/mL (range)		171 500 (23 000-399 000)
*IGHV*	Unmutated	50.3%
Binet stage	A	65.9%
	B	23.2%
	C	10.9%
LDH	Normal	81.6%
	High	18.4%
b_2_microglobulin	Normal	55.9%
	High	44.1%
Bone marrow pattern	Diffuse	41.9%
	Other	58.1%
Hepatomegaly	Yes	10.5%
	No	89.5%
Splenomegaly	Yes	26.5%
	No	73.5%
B symptoms	Yes	13.5%
	No	86.5%
Dead during follow-up	Yes	21.6%
	No	78.4%
Therapy during follow-up	Yes	45.7%
	No	78.4%

Results expressed as median or percentages.

*IGHV*: immunoglobulin heavy variable gene; LDH: lactate dehydrogenase.

### Cells and culture conditions

The human cell lines NCI-H929 and MM1S were acquired from the ATCC (American Type Culture Collection). Cell lines identity was confirmed periodically by STR analysis, PowerPlex 16 HS System kit (www.promega.com) and online STR matching analysis (www.dsmz.de/fp/cgi-bin/str.html). The human STR profile database includes data sets of 2455 cell lines from ATCC, DSMZ, JCRB and RIKEN. Both cell lines were cultured in RPMI 1640 medium supplemented with 10% of fetal bovine serum and antibiotics (Gibco). Cells were routinely checked for the presence of mycoplasma with MycoAlert kit (Lonza GmBH) and only mycoplasma-free cells were used in the experiments. The phenotypic and cytogenetic identities of the cell lines were verified by flow cytometry and FISH before the experiments.

Details on collection and preparation of patients and cell culture samples are available in Additional file 1: Supplementary Methods.

### Targeted sequence capture and DNA sequencing assays

We applied array-based sequence capture (Roche NimbleGen) followed by next-generation sequencing (Roche GS FLX Titanium sequencing platform) to analyze a large panel of genes of relevance in CLL (Additional file 2: Table S1) and two chromosomal regions: 13q14.3 (50043128–50382849 bp) and 17p13.1 (7500000–7535000). The genes had been selected according to published data and our previous gene expression data and included, for example *HSP90B1, TP53, ATM, PHLPP1, E2F1, RAPGEF2* and *PI3K.* Pyrosequencing assays were performed to analyze the sequence for 3′UTR region of the *HSP90B1* gene. Details of the design of the array, 454 sequencing, coverage statistics and data analysis, as well as the pyrosequencing assays are provided in the Additional file 1: Supplementary Methods and Additional file 2: Table S2. The sequencing data are uploaded to the Sequence Read Archive (SRA) (http://trace.ncbi.nlm.nih.gov/Traces/sra/) under accession number PRJNA275978. All the information is accessible with the following link http://www.ncbi.nlm.nih.gov/bioproject/275978.

### Luciferase reporter assay

HEK293 cells were transfected with 500 ng of the constructs detailed in the Additional file 1: Supplementary Methods and Additional file 2: Table S3, and cotransfected with 25 nM miRNA precursor molecule by nucleofection, using the HEK293 cell line program in the Amaxa II nucleofector system. Cells were collected 24 hours after transfection and Firefly and Renilla luciferase activities were measured using the Dual-Glo® Luciferase Assay System (Promega) according to the manufacturer’s protocol. Measurements were performed on a Tekan Infinite® F500 microplate reader. Firefly luciferase activity was normalized with respect to Renilla luciferase activity.

### Transfection with synthetic miRNAs

H929 and MM1S cell lines were transfected with Pre-miR™ miRNA precursors pre-miR-223 or pre-miR™ miRNA-negative, non-targeting control#1 (Ambion) at 50 nM concentration, using the nucleofector II system with C-16 program and Q-023 program, respectively (Amaxa). Transfection efficiency was assessed with Block-iT™ Fluorescent Oligo (Invitrogen) by flow cytometry.

### Quantitative real-time polymerase chain reaction analysis and Immunoblotting

This methodology is provided in Additional file 1: Supplementary Methods.

### Statistical analysis

Statistical analysis was performed using SPSS (v20). The two-sided Student’s *t* test was used to analyze differences between means (presented here with SD) of different experiments, based on triplicate determinations. Differences between the results of the qRT-PCR experiments with CLL patients were analyzed with the Mann-Whitney *U*. Kaplan-Meier analysis with the Log Rank test and Cox regression were used for survival analysis examining the impact of *HSP90B1* expression on OS and TFT. Chi-squared and Mann–Whitney U tests were employed when appropriate to correlate a range of biomarkers and clinical data according to rs2307842 status and *HSP90B1* expression. The results were considered statistically significant at *P* < 0.05.

## Results

### A targeted genome capture and next-generation sequencing strategy identifies a common polymorphism in 3′UTR of *HSP90B1*

Using a custom NimbleGen array we captured and sequenced 93 genes and two entire chromosomal regions of four CLL patients. The enrichment assay followed by NGS allowed the detection of over 1600 variations/sample (median 1721, range 1618–1823). All putative variants were first compared with published single nucleotide polymorphism (SNP) data (dbSNP build 130; http://www.ncbi.nlm.nih.gov/projects/SNP). Most of the variants detected were identified as known SNPs and 226 variants were present in all the patients, so these were discarded. Overall, 10% of variants detected in each sample were not previously described mutations. Seventy-three missense variations affecting 33 genes were detected. Most of the genes had one (70%) or two (12%) variations. Results are summarized in Additional file 2: Table S4.

**Figure 1:**
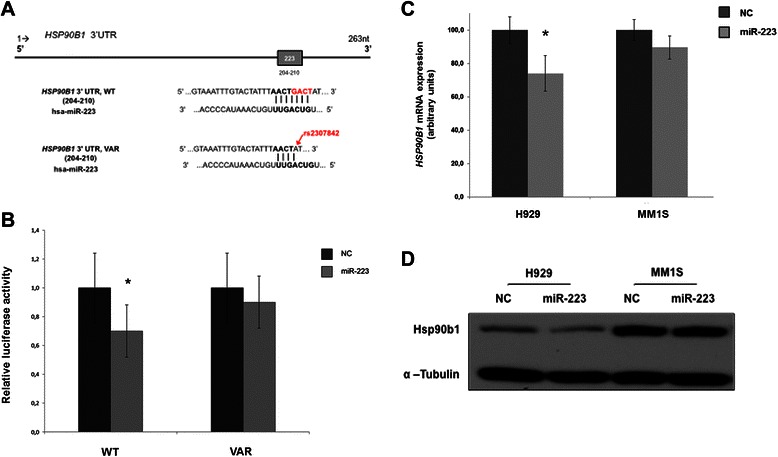
***HSP90B1*is a direct target of miR-223. (A)** 3′untranslated region (3′UTR) of *HSP90B1* (263 nt length) with a predicted binding site for miR-223 at 204–210 nt (grey box). The figure shows the mature miR-223 sequence (hsa-miR-223) aligned with *HSP90B1* 3′UTR wild type (WT, up), and with the polymorphism (VAR, below). The seed region is shown in bold. The rs2307842 polymorphism (in grey) disrupts the putative binding site for miR-223 by deleting the last three nucleotides of the seed region. **(B)** Luciferase reporter assays to confirm targeting of *HSP90B1* 3′UTR by miR-223. Ectopic miR-223 expression inhibits the wild-type but not the variant *HSP90B1* 3′UTR reporter activity in HEK293 cells. Cells were co-transfected with miR-223 precursor/negative control (NC) miRNA and with either wild-type (WT) or variant (VAR) *HSP90B1* 3′UTR reporter construct. Luciferase activity assay was performed 24 h after transfection. The columns represent normalized relative luciferase activity by means with 95% confidence intervals from 4 independent experiments (Mann–Whitney test, *P < 0.05). **(C)** and **(D)** Ectopic miR-223 expression reduced both HSP90B1 mRNA **(C)** and protein **(D)** expression in H929 cell line (WT) but not in MM1S (VAR). Cells were transfected with miR-223 precursors and negative controls. After 24 h, cells were analyzed for HSP90B1 expression by qRT-PCR **(C)** and western blot **(D)**. The data shown are representative of 3 independent experiments (Mann–Whitney test, *P < 0.05).

By applying a custom-made data analysis pipeline, we have annotated the detected variants, including reported single-nucleotide polymorphisms (SNPs), genomic location, predicted miRNA binding sites, consequences of the variant in transcripts (i.e. synonymous, missense) and protein function prediction for those variants that are predicted to result in an aminoacid sustitution. In one out of four CLL patients (25%) we identified a 4-bp insertion/deletion polymorphism (−/GACT) in 3′UTR of *HSP90B1*, filled as rs2307842 (102865778-102865781b) in the NCBI SNP database. Rs2307842 results in the deletion of four nucleotides in 3′UTR sequence, three of them being part of the predicted binding site for miR-223 (Figure 1A). According to the databases, UCSC Genome Browser, NCBI and Ensembl, the reference genome contains the ′GACT′ sequence. The major allele in the European population, according to the NCBI SNP database, is ′GACT′ (allele frequency: 0.79 ± 0.06), whereas the 4-bp deletion has a minor allele frequency of 0.21 ± 0.06. Thus, we considered the individuals carrying the ′GACT′ sequence as *wild-type* (WT) and the individuals with the 4 bp-deletion as *variants* (VAR). We hypothesized that this deletion disrupts the binding site for miR-223, thereby increasing the translation of *HSP90B1*.

### *HSP90B1* is a direct target gene of miR-223

We have confirmed that miR-223 regulates *HSP90B1* expression by 3′UTR reporter assays. First, the double-stranded oligonucleotides, corresponding to the wild-type (WT-3’UTR) or variant (VAR-3’UTR) miR-223 binding site in the 3′UTR of *HSP90B1* (NM_003299), were synthesized. PmirGLO Vectors made up of an SV40 promoter, the Renilla luciferase gene, and the 3′UTRs of *HSP90B1* were transfected into HEK293 cells along with miR-223 or negative control (NC) mimics. Relative luciferase activity was measured at 24 h. The relative luciferase activity of the construct with wild-type 3′UTR was significantly repressed following miR-223 transfection (*P* < 0.05) (Figure 1B). However, the presence of rs2307842 polymorphism in 3′UTR of *HSP90B1* (VAR-3′UTR) abolished this suppression (Figure 1B), suggesting that miR-223 directly binds to this site.

We also validated *HSP90B1* as a target gene of miR-223 by transfecting MM1S and H929 cell lines with miR-223/NC mimics and then measuring HSP90B1 expression by qRT-PCR and western blot. Sequencing assays showed that H929 cell line has WT-3′UTR, whereas rs2307842 polymorphism was present in *HSP90B1* 3′UTR of MM1S cell line (VAR-3′UTR). All experiments were done in triplicate. Exogenous expression of miR-223 downregulated the expression levels of HSP90B1 in H929 cell line (WT-3′UTR) in both mRNA (*P <* 0.05) and protein levels (Figure 1C and D). By contrast, HSP90B1 expression was not modified in the MM1S cell line (VAR-3′UTR) (Figure 1C and D). Taken together, all these results demonstrate that *HSP90B1* is a *bona fide* target gene of miR-223 and that the rs2307842 polymorphism abolishes the miR-223 regulation on HSP90B1 expression.

### rs2307842 is a common polymorphism in CLL patients

To determine the clinical impact of *HSP90B1* 3′UTR polymorphism in CLL, we screened 165 additional patients with CLL and 32 healthy controls for this polymorphism by pyrosequencing. A total of 50 paired DNA samples (CD19+ and non-CD19+ fraction cells) immunomagnetically purified from CLL patients showed complete concordance in their 3′UTR sequence, confirming that rs2307842 was the result of a SNP and not an acquired mutation. The polymorphism was found at a similar frequency in CLLs and healthy controls: 41/169 (24%) in CLL patients and 8/32 (25%) in healthy controls. These results are consistent with the data obtained from NCBI SNP database (http://www.ncbi.nlm.nih.gov/projects/SNP). Of note, no major differences regarding clinical, biological and genetic features were found between CLLs cases with the polymorphism (VAR) and wild-type (WT) (Additional file 2: Table S5).

### miR-223 is downregulated in CLL patients with IGHV unmutated genes

In order to corroborate the down-regulation of miR-223 previously reported in CLL patients with IGHV unmutated (UM) genes, 53 samples were subjected to miRNA Taqman qRT-PCR to measure miR-223 expression according to IGHV mutation status. As expected, miR-223 was downregulated in UM CLL patients when compared to mutated IGHV cases (*P* = 0.036).

### *HSP90B1* overexpression is observed in B lymphocytes from CLL patients with the rs2307842 polymorphism and IGHV-unmutated status

To test the hypothesis that *HSP90B1* overexpression may be due to a defective miR-223 regulation in CLL patients, we analyzed *HSP90B1* expression in a subgroup of patients previously characterized for the presence of the polymorphism and IGHV mutation status.

**Figure 2:**
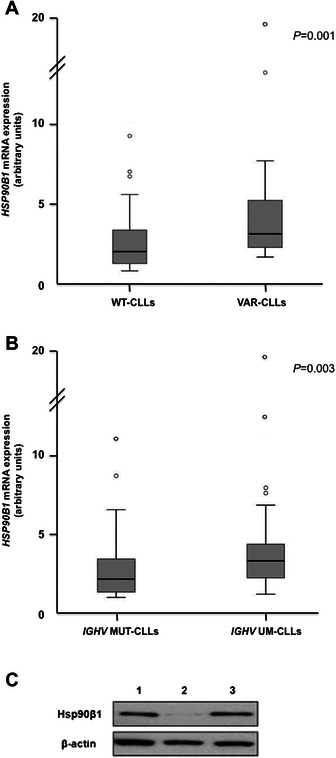
**Hsp90b1 is upregulated in CLL patients with the rs2307842 polymorphism and*IGHV*-unmutated status, as assessed by qRT-PCR and western blot analysis.** Box plots show the relative upregulation of *HSP90B1* mRNA in CLL patients with **(A)** rs2307842 (VAR) and **(B)** IGHV unmutated genes (UM) compared with wild-type CLL patients (WT) and the mutated cases (MUT), respectively. The thick line inside the box plot indicates the median expression levels and the box shows the 25th and 75th percentiles, while the whiskers show the maximum and minimum values. Outliers are represented by open circles. Statistical significance was determined by the Mann–Whitney *U* test (*P* < 0.05). **(C)** Representative lysates of purified B lymphocytes from CLL patients were prepared and Hsp90b1 protein levels were analyzed by western blot. B-actin served as loading control. Representative blots from three CLL patients are shown: #1 patient with IGHV unmutated genes (UM CLL), #2 wild-type for rs2307842 and with IGHV mutated genes (WT&MUT CLL) and #3 patient with rs2307842 (VAR CLL).

We have performed qRT-PCR in a total of 97 CLL samples: 25 out of them were CLL patients with rs2307842 (VAR-CLLs) and 72 were wild-type (WT-CLLs). qRT-PCR results showed that *HSP90B1* was overexpressed in VAR-CLLs (*P* = 0.001) (Figure 2A). To gain insight into its influence on gene expression, we have measured HSP90B1 mRNA levels in the paired normal fraction of 50 cases (13 VAR-CLLs and 37 WT-CLLs). As expected, the results showed that B lymphocytes (tumor fraction) from VAR-CLLs showed a higher level of *HSP90B1* expression than B lymphocytes from WT-CLLs (*P* = 0.001), and also from the normal cells from the same patients (VAR-CLLs) (*P* < 0.001) (Additional file 3: Figure S1). However, no changes in HSP90B1 mRNA expression were observed between tumor and normal fractions in CLLs without the SNP (P = 0.201). Thus, rs2307842 influenced *HSP90B1* overexpression only in the tumor fraction of the CLL patients with the polymorphism. Of note, we also observed overexpression of *HSP90B1* in patients with Figure 2B). The overexpression was also confirmed in the tumor fraction of the purified paired samples (data not shown).IGHV unmutated genes (UM-CLLs, n = 52) in comparison with mutated cases (MUT-CLLs, n = 45) (*P* = 0.003) (Figure 2B.

Hsp90b1 protein expression was also measured by Western blot analysis in the B lymphocytes from CLL patients harboring the variant, unmutated IGVH genes and wild-type CLLs (Figure 2C). As expected, Hsp90b1 expression was higher in CLL with *HSP90B1* the SNP and in unmutated CLL.

### *HSP90B1* overexpression is associated with a shorter time to treatment

The relationship between clinical and biological characteristics of CLL patients and HSP90B1 gene expression was analyzed. A higher *HSP90B1* mRNA *e*xpression was correlated with the presence of rs2307842 (*P* =0.003), unmutated status of the *IGHV* gene (P = 0.008) and need for treatment (P = 0.001) compared to that of patients with lower HSP90B1 mRNA expression levels.

**Figure 3:**
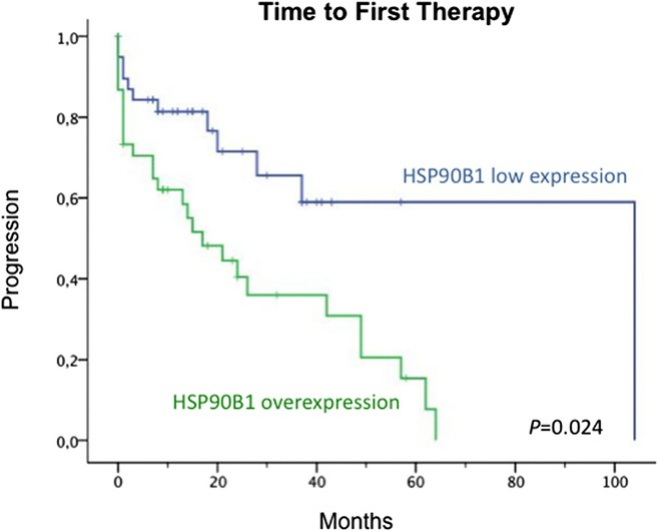
**Kaplan-Meier plot of time to first therapy of CLL patients according to*HSP90B1*expression.** Patients overexpressing HSP90B1 (green line) had a significantly shorter TFT (median = 17 months; 95%CI: 5–28.9 months) as compared to that of patients with lower HSP90B1 expression levels (blue line) (median = 104 months, *P* = 0.024).

A significantly shorter time to first therapy (TFT) was observed in the patients with *HSP90B1* overexpression (median of 17 months; 95% CI: 5–28.9 months) as compared to those cases without *HSP90B1* overexpression (median of 104 months) (p = 0.024) (Figure 3). Thus, 71% of patients in the group with *HSP90B1* overexpression required treatment vs. 31% of patients in the non-overexpressed group. Other variables associated with shorter TFT were age, non-mutated IGHV, lymphocyte count, adverse cytogenetics and the presence of B symptoms (Table 2). Multivariate analysis selected *HSP90B1* overexpression as an independent risk factor of TFT (HR: 2.63; 95% CI: 1.15-5.98; P = 0.021), after adjusting for IGHV mutation status, lymphocyte count (< vs >30000), cytogenetics (good prognosis *vs* high-risk), age (< *vs* > 65 years) and the presence of B symptoms.

## Discussion

MicroRNAs are known to inhibit gene expression by binding to the 3′UTR of the target transcript. In the present study *HSP90B1* was validated as a miR-223 direct target by 3′UTR reporter assays and transfection with synthetic miR-223 (Figure 1B and D). Thus HSP90B1 was overexpressed in CLL patients harboring unmutated *IGHV* genes and rs2307842, a common polymorphism located in *HSP90B1* 3′UTR, which disrupts the binding site of miR-223. More importantly, *HSP90B1* overexpression was independently predictive of shorter time to the first therapy. We propose that this overexpression could represent a pathogenic mechanism for miR-223 in CLL.

Functional polymorphisms in 3′UTRs of several genes (also known as miRSNPs or miR-polymorphisms) are associated with diseases affecting gene expression. Loss of microRNA function due to defective miRNA-mRNA binding results in overexpression of the target mRNA, which can be involved in key biological processes, oncogenic mechanisms or drug resistance [33-36]. Moreover, the presence of some SNPs has been suggested to influence disease progression and clinical outcome in CLL [37-42], although the results are discrepant [43-46]. Our results showed that the presence of rs2307842, a common polymorphism located in the 3′UTR of *HSP90B1* (Figure 1A), alters the interaction between the target site in *HSP90B1* and miR-223 in CLL, resulting in HSP90B1 overexpression (Figure 2A). However, no major differences regarding clinical, biological and genetic features were found between CLLs harbouring rs2307842 and wild-type cases (Additional file 2: Table S5).

We have also performed qRT-PCR using CD19+ peripheral blood lymphocytes from CLL patients displaying the polymorphism and wild-type cases (Additional file 3: Figure S1). As expected, B lymphocytes from CLL patients with the polymorphism had higher levels of *HSP90B1* than B lymphocytes from wild-type CLL patients. Surprisingly, non-clonal cells from CLL patients with the polymorphism showed levels of HSP90B1 mRNA similar to that of wild-type CLL patients (both CD19+ and non-CD19+ fraction cells). These findings suggest that a regulatory mechanism of *HSP90B1* expression could be present in cells with rs2307842. Further work is needed to understand the relevance and functional consequences of this common polymorphism in CLL patients. Of note, our study shows that the presence of variants that alter the 3′UTR-site targeted by the miRNA could be an alternative mechanism to the presence of mutations inside or surrounding microRNA genetic loci.

Although miR-223 has been related to HSP90 in osteosarcoma [47], miR-223 function is not well characterized in CLL. However the expression levels significantly decrease with the progression of the disease and miR-223 downregulation has been associated with higher tumor burden, disease aggressiveness, and poor prognostic factors, such as IGHV unmutated genes (UM CLL) [8,13,14]. Despite the proven implication of miR-223 expression in CLL prognosis, little is known about the molecular mechanisms that may be responsible for the poor outcome of CLL patients showing miR-223 downregulation and, unlike other miRNAs with prognostic value in CLL, such as miR-181b and miR-29c, the target of miR-223 in CLL is still unknown [48,49]. Our results confirmed the down-regulation of miR-223 in IGHV UM CLLs. Moreover, the present results, demonstrating that *HSP90B1* is a direct target gene of miR-223, provide more information about how the downregulation of miR-223 could determine the poor outcome of IGHV UM CLLs, possibly by upregulation of *HSP90B1* expression (Figure 2B and C). Limited data are available regarding the expression of HSP90 in CLL. In myelodisplastic syndromes, high levels of HSP90 were associated with shorter survival and increased risk of progression into acute myeloid leukemia (AML) [50,51]. In AML, the percentage of HSP90-positive cells was correlated with that of Bcl2-positive cells and higher expression of HSPs was associated with lower complete remission rate and poor survival [52,53]. Of note, we also observed a correlation between *HSP90B1* and *BCL2* overexpression in CLL patients (data not shown). HSP90 has been proposed to have a role in the modulation of apoptosis and is implicated in the resistance of leukemic cells to chemotherapeutic agents and recent evidence suggests that HSP90 inhibitors such as 17-AAG and 17-DMAG [23], which have shown preclinical efficacy, could be a therapeutic option in CLL [25]. More importantly, our data suggest that HSP90B1 overexpression is independently predictive of shorter time to first therapy in CLL (Table 2).

**Table 2:** **Univariate and multivariate analysis for time to first therapy (TFT) in this series**

			Univariate analysis				Multivariate analysis			
Characteristics	Events	Total	Median	LCI	UCI	*P*	HR	LCI	UCI	*P*
HSP9081 expression										
Normal	12	39	104.0	-	-	-	-	-	-	-
High	28	39	17.0	5.0	28.9	0.024	2.7	1.18	6.46	0.026
IGVH identity										
<98%	15	60	104.0	11.3	196.7	-	-	-	-	-
≥98%	39	57	14.0	6.8	21.2	<0.001	2.34	1.03	5.35	0.043
Lymphocyte										
<30000	35	90	53.0	35.1	70.8	-	-	-	-	-
≥30000	25	37	8.0	0.0	17.5	<0.001	4.2	1.75	10.05	0.001
Cytogenetics										
Good prognosis	28	82	57.0	38.3	75.7	-	-	-	-	-
Poor prognosis	16	21	9.0	1.7	16.2	<0.001	1.65	1.907	2.54	0.023
Age (years)										
≥65	29	71	42.0	18.3	65.7	-	-	-	-	-
<65	29	53	24.0	6.6	41.4	0.04	0.37	0.17	0.83	0.015
B symptoms										
No	42	194	49.0	34.3	63.7	-	-	-	-	-
Yes	15	18	1.0	0.0	2.1	<0..001	0.17	0.06	0.53	0.002

*IGVH*: immunogllobulin heavy variable gene; LCI: 95% lower confidence interval; UCI: 95% upper confidence interval; HR: Hazard ratio.

Time to first theraphy (TFT) was defined as the interval between diagnosis and the treatment.

## Conclusions

Our study highlights the relevance of miRNAs as critical players in the pathogenesis of CLL and shows for the first time that miR-223 modulates HSP90B1 expression in B lymphocytes of CLL. These results provide a plausible explanation of why CLL patients harboring miR-223 downregulation are associated with a poor outcome. Our work also points out HSP90B1 overexpression as a new pathogenic mechanism in CLL and a promising therapeutic target, at least in a subgroup of CLL patients.

## Additional files

Additional file 1:
**Supplementary Method.**

Additional file 2: Tables S1-S5. ᅟ
Additional file 3: Figure S1. Differential expression of *HSP90B1* in purified paired samples (CD19+ and non- CD19) from CLL patients assessed by qRT-PCR analysis. Box plots show the relative upregulation of *HSP90B1* in B lymphocytes (CD19+ fraction cell, *CD19+*) from CLL patients with rs2307842 (VAR-CLLs) compared with the non- CD19+ fraction cell (*non-CD19*) from the same patients (*P*<0.001) and the wild-type CLL patients (WT-CLLs) (*P*=0.001). The thick line inside the box plot indicates the median expression levels and the box shows the 25th and 75th percentiles, while the whiskers show the maximum and minimum values. Outliers are represented by open circles. Statistical significance was determined by the Mann-Whitney *U* test (*P*<0.05).

## Abbreviations

3′UTR: 3′untranslated region
ATCC: American Type Culture Collection
Bp: Base pair
CI: Confidence interval
CLL: Chronic lymphocytic leukemia
FISH: Fluorescence in situ hybridization
HR: Hazard ratio
HSP: Heat shock protein
IGHV: Immunoglobulin heavy chain variable
MiRNA: MicroRNA
MUT: Mutated
NCI: National Cancer Institute
NGS: Next generation sequencing
OS: Overall survival
SNP: Single nucleotide polymorphism
TFT: Time to first therapy
UM: Unmutated
VAR: Variant
Vs: Versus
WT: Wild type
