# Supplementary material for: MicroRNA-223 is a novel negative regulator of HSP90B1 in CLL
Source: BMC Cancer. 2015 Apr 8;15:238. doi: 10.1186/s12885-015-1212-2 (PMC4404064; doi:10.1186/s12885-015-1212-2)
Supplement: Additional file 1: — Supplementary Method. [file 12885_2015_1212_MOESM1_ESM.pdf]

SUPPLEMENTARY METHODS

Patients and controls

Four CLL patients were selected for the targeted sequence capture and DNA sequencing assay. These patients comprised three males and one female aged 41 to 66 years at diagnosis. The *IGHV* gene was unmutated in two patients and mutated (<98% homology) in the other two. Two patients had a 13q deletion, one had an 11q deletion and one showed no cytogenetic aberrations with FISH.

In the 169 patients cohort, the median age was 66 (range, 34 to 90 years). Most patients were male (66%) and were in Binet clinical stage A (65.9%), while 23.2% were in stage B and the remaining 10.9% in stage C. The study was approved by the local ethics committees.

Collection and preparation of samples

Peripheral blood mononuclear cells (PBMCs) from all CLL patients were isolated by Ficoll–Hypaque gradient centrifugation (Amersham Biosciences), snap-frozen and stored at -80°C. CLL B lymphocytes were positively purified using magnetically activated cell sorting (MACS) CD19 MicroBeads (Miltenyi Biotec). CD19 selection resulted in >98% purity, as determined by flow cytometry.

DNA was extracted from fresh-frozen samples using a Qiagen kit. To ensure good quality, DNA was measured using NanoDrop ND-1000 (ND-1000; NanoDrop Technologies). Only samples with OD 260 nm/280 nm >1.8 were included. The integrity of the DNA was visually inspected on a 1% agarose gel. RNA was extracted using Trizol reagent (Invitrogen) according to the standard protocol. RNA integrity was assessed using Agilent 2100 Bioanalyzer (Agilent Technologies).

Targeted sequence capture and DNA sequencing assays

1. NimbleGen Target-Region Capture

A custom Sequence Capture 385K Human Array was designed and manufactured by Roche NimbleGen. A total of 385,000 unique, overlapping probes 60–90 nucleotides in length were designed that included all known exons and untranslated regions (UTRs). In total, 1564 exons from a large panel of genes, including relevant genes in CLL and two target regions 13q14.3: 50043128-50382849 bp and 17p13.1: 7500000-7535000; NCBI build 36.1, hg18) were selected, targeting 845212 bases. Approximately 5 µg of genomic DNA from 4 CLL patients were fragmented to a size range of 300–500 base pairs (bp) with the use of a GS Nebulizer Kit (Roche Applied Science) to generate blunt-ended fragments. The fragmented DNA was purified (DNA Clean & Concentrator-25, Zymo Research) and analyzed on an Agilent Bioanalyzer 2100 DNA Chip 7500 according to the manufacturer's instructions. The fragmented DNA was then processed according to the recommended NimbleGen protocol (Roche Applied Science, User Guide 3.1; July 2008). In brief, linkers were ligated to the polished fragments in the library to provide a priming site for post-enrichment amplification of the eluted fragment pool. The linker-terminated fragments were then denatured to produce single-stranded products. The resulting library was hybridized to a custom 385K array for 72 h at 42ºC,

with the use of the NimbleGen Sequence Capture Hybridization System 4. The hybridized DNA from the target regions was washed and eluted with the use of a NimbleGen Wash and Elution Kit according to the manufacturer's instructions. The eluted sample was amplified by ligation-mediated PCR with the use of primers complementary to the sequence of the adaptors.

2. 454 Sequencing

We applied NGS technology using 454 FLX Titanium chemistry according to the manufacturer's protocols (Roche Applied Science).[1] Sequencing-compatible linkers were ligated to the eluted samples from the capture microarrays. The libraries were subsequently diluted, amplified on beads using emulsion PCR and sequenced using the 454 FLX sequencing instrument.

3. Sequencing data analysis

Basic raw data analysis was carried out using the GS Run Browser and GS Reference Mapper software version 2.0.01 (Roche Applied Science). Following in silico removal of the linker sequence, each sequence read was in comparison with the entire appropriate version of the human genome. Captured sequences mapped uniquely back to regions within the target regions were considered sequencing hits. These were then used to calculate the percentage of reads that did hit target regions, and the n-fold sequencing coverage for the entire target region. All putative variances were first compared with published single nucleotide polymorphism (SNP) data (dbSNP build 130; http://www.ncbi.nlm.nih.gov/projects/SNP). We used a custom-made data analysis pipeline to annotate detected variants with various kinds of information.

4. Coverage statistics

According to NCBI build 36.1, hg18 reference genome, the final target bases covering the 93 target genes and target regions 13q14.3 and 17p13.1 were defined to be 845 212 bp; of those 750 594, target bases (99.39%) were covered by capture oligonucleotides as defined by NimbleGen's default settings for probe selection. 5 134 bp (0.6%) of the initial target region were omitted due to reasons of specificity and uniqueness (**Supplementary Table S2**). This was sufficient to reach an average 21.7-fold target coverage per individual.

Pyrosequencing assays

The following oligonucleotide primers were used to amplify a 170-bp genomic fragment spanning the sequence for 3´UTR region of the *HSP90B1* gene: *HSP90B1*-For_Bio: 5-CTGCACTGTAAAATGTGGGATTAT-3 and *HSP90B1*-Rev: 5-AGACACTGAGTATTTGGGATCTTT-3. The 5-ends of the forward primer were conjugated with biotin (Bio). PCR was performed using a DNA thermal cycler (Applied Biosystem, ABI 9700). The template was denatured initially for 5 min at 96°C followed by 40 amplification cycles containing: initial denaturation at 95°C for 30 s, followed by annealing for 30 seconds at 60°C and extension at 72°C for 30 s. Final extension was done at 72°C for 5 min. The amplified PCR products were checked by electrophoresis on 1% agarose gels and stored at -20ºC.

Pyrosequencing was carried out on a PyroMark Q24 system (Qiagen) according to the manufacturer's protocol. The pyrosequencing primer *HSP90B1*-Py1-5-TGACAAGATTTTACATCA-3 was used with the nucleotide dispensation order: CAGAGTAGTCA. A total of 10 μl PCR product, 2 μl Streptavidin Sepharose High Performance beads (GE Healthcare Bio-Sciences AB, Sweden, Uppsala), 28 μl water and 40 μl binding buffer (Qiagen) were mixed and agitated constantly for 10 min at 1,400 rpm. The PCR products attached to the beads were washed in 70% ethanol,

followed by denaturation in 0.2 N NaOH and washing buffer (Qiagen). Purified DNA samples were annealed to the sequencing primer (0.3 μM) in 25 μl annealing buffer (Qiagen) and denatured for 2 min at 80ºC, followed by cooling to room temperature for 5 min. The samples were then processed in the PyroMark Q24 Instrument, with a running time of 15 min for 24 samples. Setup of assay and sequence-run as well as analysis were performed using the PyroMark Q24 Software.

Luciferase reporter assay: constructs

The double-stranded oligonucleotides corresponding to the wild-type (WT-3'UTR) or variant (VAR-3'UTR) miR-223 binding site in the 3´UTR of *HSP90B1* (NM_003299) were synthesized (Sigma-Aldrich) and ligated between the PmeI and XbaI restriction sites of the pmirGLO vector (Promega). The variant (VAR-3'UTR) miR-223 binding site was generated based on *HSP90B1* 3´UTR sequence in which 4 nucleotides were deleted at the miR-223 seeding region, corresponding to the polymorphism found in the Targeted Sequence Capture and DNA Sequencing assay (rs2307842). The oligonucleotides sequences are presented in **Supplementary Table S3**.

Quantitative real-time polymerase chain reaction analysis

To detect mature miR-223 expression levels, a TaqMan quantitative real-time polymerase chain reaction (qRT-PCR miRNA) assay (Applied Biosystem) was performed. The relative expression of mature miR-223 levels normalized to the RNU43 endogenous control was determined using the $2^{-\Delta Ct}$ method. Each measurement was performed in duplicate.

To detect the mRNA expression of HSP90B1, total RNA (1 μg) was reverse-transcribed to cDNA using SuperScript™ III First-Strand Synthesis SuperMix (Invitrogen). SYBR Green qRT-PCR was done in triplicate with an iQ™SYBR® Green Supermix kit (Bio-Rad) using the IQ5 Multicolor Real-Time PCR Detection System (Bio-Rad) with the following gene-specific primers: *GAPDH,* forward 5´-CAGGGCTGCTTTTAACTCTGG-3´and reverse 5´-GGGTGGAATCATATTGGAACA-3´and *HSP90B1,* forward 5´-CCGAAGAAGAACCTGAAGAG-3´and reverse 5´-CATCTGTTCCCACATCCATT-3´. The *GAPDH* gene was used as the internal control and expression data were analyzed by the comparative Ct method. For analytical purposes, we adopted cut-off values according to median-expression levels for HSP90B1.

Immunoblotting

Whole-cell lysates were collected using RIPA buffer (Sigma-Aldrich) containing protease inhibitors (Complete Protease Inhibitor Cocktail Tablets, Roche) and phosphatase inhibitors (Phosphatase Inhibitor Cocktail A and B, Santa Cruz Biotechnology). Protein concentration was measured using the Bradford assay (Bio-Rad). Protein samples (40 μg/lane) were subjected to sodium dodecyl sulfate-polyacrylamide gel electrophoresis (SDS-PAGE) and transferred to 0.45 μm polyvinylidene fluoride (PVDF) membrane (Bio-Rad). The primary antibodies used for immunoblotting were anti-Hsp90b1 (Cell Signalling Technology Inc.) and anti-β-actin (Sigma-Aldrich) as an internal control for protein loading. The membranes were then washed and incubated with the secondary horseradish peroxidase-linked anti-mouse IgG and anti-rabbit IgG antibodies (PierceNet) (1:10000). Chemiluminescence was detected using Amersham ECL Plus™ Western Blotting Detection Reagent (GE Healthcare).

Reference List

1. Margulies M, Egholm M, Altman WE et al. Genome sequencing in microfabricated high-density picolitre reactors. Nature 2005;437:376-380.
