# Supplementary material for: MicroRNA-223 is a novel negative regulator of HSP90B1 in CLL
Source: BMC Cancer. 2015 Apr 8;15:238. doi: 10.1186/s12885-015-1212-2 (PMC4404064; doi:10.1186/s12885-015-1212-2)
Supplement: Additional file 2: Tables S1-S5. — ᅟ [file 12885_2015_1212_MOESM2_ESM.pdf]

Table S1. **Genes targeted by sequence capture.**

This table contains the gene symbol, the common gene name, the chromosome, the total number of exons, the transcript ID of the largest isoform and the gene description.

Gene Symbol	Gene Name	Chr	Exon count	RefSeq ID	Gene description
CLIC4	uc001bjn.1	chr1	7	NM_013943	chloride intracellular channel 4
HAX1	uc001fes.1	chr1	7	NM_006118	HCLS1 associated protein X-1 isoform a
MRPL37	uc001cxb.1	chr1	7	NM_016491	mitochondrial ribosomal protein L37
UROD	uc001cna.1	chr1	10	NM_000374	uroporphyrinogen decarboxylase
FCRL1	uc001frg.1	chr1	11	NM_052938	Fc receptor-like 1
NRAS	uc009wgu.1	chr1	7	NM_002524	neuroblastoma RAS viral (v-ras) oncogene
CXCR4	uc002tuz.1	chr2	2	NM_003467	chemokine (C-X-C motif) receptor 4 isoform b
SLC16A14	uc002vqd.1	chr2	5	NM_152527	solute carrier family 16 (monocarboxylic acid
HSPD1	uc002uuk.1	chr2	12	NM_199440	chaperonin
HTRA2	uc002smn.1	chr2	9	NM_013247	HtrA serine peptidase 2 isoform 1 preproprotein
MRPL35	uc002srf.2	chr2	5	NM_145644	mitochondrial ribosomal protein L35 isoform b
MRPS9	uc002tcn.2	chr2	11	NM_182640	mitochondrial ribosomal protein S9
ABCB6	uc002vkc.1	chr2	19	NM_005689	ATP-binding cassette, sub-family B, member 6
ALAS1	uc003dcz.1	chr3	11	NM_199166	5-aminolevulinate synthase 1
PCCB	uc003eqy.1	chr3	15	NM_000532	propionyl Coenzyme A carboxylase, beta
BCL6	uc003frp.2	chr3	10	NM_001706	B-cell lymphoma 6 protein isoform 1
CAMP	uc003csj.1	chr3	4	NM_004345	cathelicidin antimicrobial peptide
FANCD2	uc003buy.1	chr3	44	NM_001018115	Fanconi anemia complementation group D2 isoform
MLF1	uc003fbx.1	chr3	9	NM_022443	myeloid leukemia factor 1 isoform 1
PIK3CA	uc003fjk.1	chr3	21	NM_006218	phosphoinositide-3-kinase, catalytic, alpha
PIK3CB	uc003esu.1	chr3	22	NM_006219	phosphoinositide-3-kinase, catalytic, beta
SLC25A38	uc003cjo.2	chr3	7	NM_017875	solute carrier family 25, member 38
VHL	uc003bvc.1	chr3	3	NM_000551	von Hippel-Lindau tumor suppressor isoform 1
CCNA2	uc003iec.2	chr4	8	NM_001237	cyclin A
RAPGEF2	uc003iqg.2	chr4	24	NM_014247	Rap guanine nucleotide exchange factor 2
TET2	uc003hxk.2	chr4	11	NM_001127208	tet oncogene family member 2 isoform a

HSPA4	uc003kyj.1	chr5	19	NM_002154	heat shock 70kDa protein 4
CCNB1	uc003jvm.1	chr5	9	NM_031966	cyclin B1
CTNNA1	uc003ldh.1	chr5	18	NM_001903	catenin, alpha 1
MRPL18	uc003qsw.2	chr6	4	NM_014161	mitochondrial ribosomal protein L18
E2F3	uc003nda.2	chr6	7	NM_001949	E2F transcription factor 3
ESR1	uc003qom.2	chr6	10	NM_001122742	estrogen receptor alpha isoform 4
SYNE1	uc003qou.2	chr6	146	NM_182961	spectrin repeat containing, nuclear envelope 1
CDK6	uc003ume.1	chr7	9	NM_001259	cyclin-dependent kinase 6
CLIP2	uc003uam.1	chr7	17	NM_003388	CAP-GLY domain containing linker protein 2
CUX1	uc003uys.2	chr7	24	NM_181552	cut-like homeobox 1 isoform a
MLL3	uc003wla.1	chr7	59	NM_170606	myeloid/lymphoid or mixed-lineage leukemia 3
PIK3CG	uc003vdw.1	chr7	11	NM_002649	phosphoinositide-3-kinase, catalytic, gamma
MTFR1	uc003xvo.1	chr8	7	NM_014637	mitochondrial fission regulator 1
CCNE2	uc003yhd.1	chr8	12	NM_057749	cyclin E2
SLC25A37	uc003xdo.1	chr8	4	NM_016612	solute carrier family 25, member 37
ALAD	uc004bhm.2	chr9	12	NM_000031	delta-aminolevulinic acid dehydratase
FXN	uc004aha.1	chr9	5	NM_000144	frataxin isoform 1 preproprotein
MRPL41	uc004cnh.2	chr9	2	NM_032477	mitochondrial ribosomal protein L41
STOML2	uc003zwi.1	chr9	10	NM_013442	stomatin (EPB72)-like 2
ENG	uc004bsj.2	chr9	15	NM_001114753	endoglin isoform 1 precursor
JAK2	uc003ziw.1	chr9	25	NM_004972	Janus kinase 2
TUSC1	uc003zpx.1	chr9	1	NM_001004125	tumor suppressor candidate 1
UROS	uc001lix.2	chr10	10	NM_000375	uroporphyrinogen III synthase
CDC2	uc001jlf.1	chr10	8	NM_001786	cell division cycle 2 isoform 1
CUGBP2	uc001ikp.2	chr10	15	NM_001025076	CUG triplet repeat, RNA binding protein 2
FAS	uc001kfr.1	chr10	9	NM_000043	tumor necrosis factor receptor superfamily,
VCL	uc001jwd.1	chr10	22	NM_014000	vinculin isoform meta-VCL
ACAT1	uc001pjy.1	chr11	12	NM_000019	acetyl-Coenzyme A acetyltransferase 1 precursor
ATM	uc001pkg.1	chr11	22	NM_138292	ataxia telangiectasia mutated protein isoform 2
ATM	uc009yxr.1	chr11	64	NM_000051	ataxia telangiectasia mutated protein isoform 1
CAPRIN2	uc001rji.1	chr12	18	NM_001002259	C1q domain containing 1 isoform 1
MRPL42	uc001tcq.1	chr12	7	NM_172178	mitochondrial ribosomal protein L42 isoform b

MTERFD3	uc001tmg.1	chr12	3	NM_025198	transcription termination factor-like protein
SHMT2	uc001snf.1	chr12	12	NM_005412	serine hydroxymethyltransferase 2
CDK2	uc001sit.2	chr12	7	NM_001798	cyclin-dependent kinase 2 isoform 1
CHFR	uc001uld.1	chr12	18	NM_018223	checkpoint with forkhead and ring finger
HSP90B1	uc001tkb.1	chr12	18	NM_003299	heat shock protein 90kDa beta, member 1
FLT3	uc001urw.1	chr13	24	NM_004119	fms-related tyrosine kinase 3
ARG2	uc001xjs.1	chr14	8	NM_001172	arginase 2 precursor
PCK2	uc001wlr.1	chr14	10	NM_001018073	mitochondrial phosphoenolpyruvate carboxykinase
DICER1	uc001ydw.2	chr14	28	NM_030621	dicer1
TCL1A	uc001yfb.2	chr14	4	NM_021966	T-cell leukemia/lymphoma 1A
CCNB2	uc002afz.1	chr15	9	NM_004701	cyclin B2
IQGAP1	uc002bpl.1	chr15	38	NM_003870	IQ motif containing GTPase activating protein 1
CDH13	uc002fgx.1	chr16	14	NM_001257	cadherin 13 preproprotein
PHLPPL	uc002fax.1	chr16	18	NM_015020	PH domain and leucine rich repeat protein
MRPL27	uc002iqq.1	chr17	4	NM_016504	mitochondrial ribosomal protein L27
CYB5A	uc002llh.1	chr18	6	NM_001914	cytochrome b-5 isoform 2
FECH	uc002lgp.2	chr18	11	NM_001012515	ferrochelatase isoform a precursor
BCL2	uc002lit.1	chr18	3	NM_000633	B-cell lymphoma protein 2 alpha isoform
PHLPP	uc002lis.1	chr18	18	NM_194449	PH domain and leucine rich repeat protein
TIMM50	uc002olu.1	chr19	11	NM_001001563	translocase of inner mitochondrial membrane 50
CCNE1	uc002nsn.1	chr19	12	NM_001238	cyclin E1 isoform 1
CEBPA	uc002nun.1	chr19	1	NM_004364	CCAAT/enhancer binding protein alpha
CYP4F3	uc002nbj.1	chr19	13	NM_000896	cytochrome P450, family 4, subfamily F,
GDF15	uc002niv.2	chr19	2	NM_004864	growth differentiation factor 15
TGM2	uc002xhr.1	chr20	13	NM_004613	transglutaminase 2 isoform a
E2F1	uc002wzu.2	chr20	7	NM_005225	E2F transcription factor 1
GART	uc002yrx.1	chr21	22	NM_000819	phosphoribosylglycinamide formyltransferase,
SOD1	uc002ypa.1	chr21	5	NM_000454	superoxide dismutase 1, soluble
ADAMTS1	uc002ymf.1	chr21	9	NM_006988	ADAM metallopeptidase with thrombospondin type 1
GCAT	uc003aua.1	chr22	10	NM_014291	glycine C-acetyltransferase precursor
TIMP3	uc003anb.1	chr22	5	NM_000362	tissue inhibitor of metalloproteinase 3
ABCB7	uc004ebz.1	chrX	16	NM_004299	ATP-binding cassette, sub-family B, member 7

AIFM1	uc004evj.1	chrX	17		Homo sapiens apoptosis-inducing factor AIF mRNA, nuclear gene encoding mitochondrial protein, complete cds.
ALAS2	uc004dua.2	chrX	11	NM_000032	5-aminolevulinate synthase 2 isoform a
APOO	uc004dax.1	chrX	9	NM_024122	apolipoprotein O

Table S2. Coverage statistics

Total number of target regions	1266
Total region bases	845212
Percent target regions covered	98.06
Target bases covered	750594
Percent target bases covered	99.39
Target bases not covered	51.34
Percent target bases not covered	0.6

Table S3. Oligonucleotides sequences used for the 3´UTR luciferase constructs

Oligonucleotide designation	Sequence (5´-3´)
**HSP90B1-WT**	
**Forward**	AAACTGTAAATTTGTACTATTTAACTGACTATTCTTGATGTAAAATCTTGTCT
**Reverse**	CTAGAGACAAGATTTTACATCAAGAATAGTCAGTTAAATAGTACAAATTTACAGTTT
**HSP90B1-VAR**	
**Forward**	AAACTGTAAATTTGTACTATTTAACTATTCTTGATGTAAAATCTTGTCT
**Reverse**	CTAGAGACAAGATTTTACATCAAGAATAGTTAAATAGTACAAATTTACAGTTT

Table S4. Variations detected in the targeted resequencing study

Number of variations/gen	Genes		
	n	%	Gene Symbol
1	23	70	ABCB6, ADAMTS1, ALAS2, BCL6, CAPRIN2, CDK6, CYP4F3, DICER1, DLEU7, FECH, GART, HSPD1, MLF1, MLL3, MRLPL27, MRLPL35, MRLPL37, MTFR1, PCCB, PIK3CA, RAPGEF2, SLC25A37, SOD1
2	4	12	ACAT1, CHFR, PHLPP2, TIMM50
3	1	3	FLT3
4	3	9	ATM, GCAT, GDF15
6	1	3	TET2
15	1	3	SYNE1

Table S5. Main clinical, biological and genetic characteristics of 169 CLL patients according to the rs2307842 status

	Category	wild type HSP90B1 (n=128)	VAR- HSP90B1 (n=41)	*P*
Gender	Male	84/128 (66%)	26/41 (63%)	0.841
Age (years), median (range)		68 (34-90)	63 (46-85)	0.054
Binet stage	A	64/99 (65%)	22/34 (65%)	0.423
	B	22/99 (22%)	10/34 (29%)	
	C	13/99 (13%)	2/34 (6%)	
White blood cells ($x10^9$/L), median (range)		23 050 (7 100-188 020)	18 500 (7 080-123 000)	**0.043**
Lymphocytes ($x10^9$/L), median (range)		15 741 (4 100-180 000)	14 430 (1 580-100 000)	0.142
Hemoglobin ($x10^9$/L), median (range)		14.0 (4.4-16.8)	14.3 (9.5-16.8)	0.069
Platelets ($x10^9$/L), median (range)		175 000 (45 000-333 000)	159 000 (23 000-380 000)	0.640
FISH	13q14 deletion	50/125 (40%)	16/41 (39%)	0.852
	Trisomy 12	29/125 (23%)	7/41 (17%)	0.515
	11q22-q23 deletion	14/125 (11%)	6/41 (15%)	0.575
	17p13 deletion	8/125 (6%)	2/41 (5%)	1.000
	Normal FISH	35/125 (28%)	13/41 (32%)	0.549
IGVH	unmutated	60/111 (54%)	14/35 (40%)	0.147
LDH	>UNL	22/98 (22%)	3/33 (9%)	0.091
b2 microglobulin	>UNL	39/91 (43%)	15/31 (48%)	0.592
LDT	<1 year	22/88 (25%)	7/30 (23%)	0.791
Bone marrow pattern	diffuse	21/50 (42%	9/21 (43%)	0.947
Hepatomegaly	Yes	11/96 (11%)	3/32 (9%)	1.000
Splenomegaly	Yes	26/97 (27%)	9/34 (26%)	0.970

B symptoms	Yes	15/96 (16%)	3/32 (9%)	0.559
Died during follow up	Yes	22/100 (22%)	8/34 (23%	0.853
Therapy during follow up	Yes	47/100 (47%)	13/33 (39%)	0.446

IGVH unmutated: ≥ 98% homology with germline; LDH: lactate dehydrogenase; UNL: upper normal level; LDT: lymphocyte doubling time

Significant *P*-values are shown in **bold.**
