# Supplementary material for: MicroRNA-223 is a novel negative regulator of HSP90B1 in CLL
Source: BMC Cancer. 2015 Apr 8;15:238. doi: 10.1186/s12885-015-1212-2 (PMC4404064; doi:10.1186/s12885-015-1212-2)
Supplement: Additional file 3: Figure S1. — Differential expression of HSP90B1 in purified paired samples (CD19+ and non- CD19) from CLL patients assessed by qRT-PCR analysis. Box plots show the relative upregulation of HSP90B1 in B lymphocytes (CD19+ fraction cell, CD19+) from CLL patients with rs2307842 (VAR-CLLs) compared with the non- CD19+ fraction cell (non-CD19) from the same patients (P<0.001) and the wild-type CLL patients (WT-CLLs) (P=0.001). The thick line inside the box plot indicates the median expression levels and the box shows the 25th and 75th percentiles, while the whiskers show the maximum and minimum values. Outliers are represented by open circles. Statistical significance was determined by the Mann-Whitney U test (P<0.05). [file 12885_2015_1212_MOESM3_ESM.pdf]

SUPPLEMENTARY FIGURE

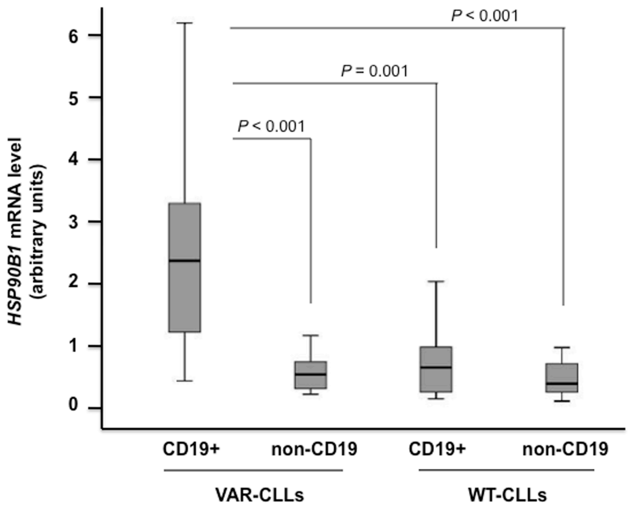

Supplementary Figure S1.

Differential expression of *HSP90B1* in purified paired samples (CD19+ and non-CD19) from CLL patients assessed by qRT-PCR analysis.
Box plots show the relative upregulation of *HSP90B1* in B lymphocytes (CD19+ fraction cell, *CD19+*) from CLL patients with rs2307842 (VAR-CLLs) compared with the non-CD19+ fraction cell (*non-CD19*) from the same patients (P<0.001) and the wild-type CLL patients (WT-CLLs) (P=0.001). The thick line inside the box plot indicates the median expression levels and the box shows the 25th and 75th percentiles, while the whiskers show the maximum and minimum values. Outliers are represented by open circles. Statistical significance was determined by the Mann-Whitney U test (P<0.05).
